# Bactericidal Effect of Different Anti-Microbial Agents on Fusobacterium Nucleatum Biofilm

**DOI:** 10.7759/cureus.1335

**Published:** 2017-06-11

**Authors:** Rupa Ashok, Arathi Ganesh, Kandaswamy Deivanayagam

**Affiliations:** 1 Department of Conservative Dentistry and Endodontics, Faculty of Dental Sciences, Sri Ramachandra University, Porur, Chennai, India

**Keywords:** fusobacterium nucleatum, turmeric solution, sodium hypochlorite, chlorhexidine, root canal irrigant

## Abstract

**Introduction:**

The root canal anatomy of the teeth is very complex. Complete debridement of the root canals is a challenge and is very important for the success of the root canal treatment. Hence, this study was done to find an effective irrigant which can be used during root canal treatment.

**Objective:**

The bactericidal effect of a potential root canal irrigant was compared with two commonly used root canal irrigants against monoculture biofilm of a commercially available isolate of Fusobacterium nucleatum.

**Methods:**

A monoculture biofilm of Fusobacterium nucleatum was grown on glass slides. The glass slides containing the biofilm were immersed in centrifuge tubes containing 5% sodium hypochlorite, 2% Chlorhexidine, 6% turmeric solution, 9% turmeric solution and distilled water for a time span of one minute. A wire loop was used to scrape off the biofilms onto sterile brain heart infusion agar plates. This was further subjected to an incubation period of 96 hours at 37° C. Colony forming units were quantified by statistical analysis and results were obtained.

**Results:**

The anti-bacterial activity of 6% and 9% turmeric solution was statistically significant against Fusobacterium nucleatum when compared to 2% Chlorhexidine and 5% sodium hypochlorite.

**Conclusion:**

In endodontic treatment, turmeric solution may be considered as an effective irrigant.

## Introduction

Root canal serves as a microenvironment for several microbial species to form dense bacterial biofilms and cause persistent infections [1]. The necrotic and infected pulp provides an ideal habitat for the microorganisms [2]. Providing a hermetic seal of the root canal space thereby averting secondary infection, while simultaneously trying to eradicate or reduce the root canal microbiota is the ultimate goal of endodontic treatment [3]. Microorganisms in the root canals occur in various forms, namely as planktonic, clusters and as biofilms. Biofilm consists of communities of microorganisms that are firmly fixed in extracellular polymeric substance and they are attached onto a surface [1,3]. Microorganisms, which are found freely suspended in an aqueous medium, are called planktons [4]. Biofilms are established by microorganisms for enhancing protection and their physiologic functions. They strongly resist the host immune response and antimicrobial agents. Bacteria within biofilms have increased potential to resist adverse conditions such as nutritional distress, acidic or alkaline environment and antimicrobials than planktonic bacteria [3]. The microorganisms in the biofilms are around more than thousand times resistant to antimicrobial agents than planktonic forms [5].

Fusobacterium nucleatum is a gram-negative, obligate anaerobic rod, non-sporing oral bacterium occurring normal flora in the mouth. It is highly pathogenic, as it produces tissue irritants and are usually found in periodontal lesions, synergistically acting with other types of bacteria [6]. Increased pathogenicity has been observed when there is combination of anaerobes like prevotella, porphyromonas and fusobacterium genera [7]. Gomes, et al. [8] reported that fusobacterium species was associated with specific signs and symptoms of endodontic origin such as pain, tenderness on percussion, wet canals, and in purulent exudates. Hence, it has become necessary to study the influence of irrigants on monoculture biofilms of Fusobacterium nucleatum to prevent oral and systemic infections.

Ability to remove organic and inorganic debris, possessing reduced toxicity, ability to lubricate effectively while simultaneously eradicating the smear layer to completely disinfect the root canal are some of the ideal requirements of an ideal irrigant [9]. Sodium hypochlorite and Chlorhexidine possess many properties of an ideal irrigant. However, previous studies have shown variations regarding the anti-microbial potential of sodium hypochlorite and Chlorhexidine [10-11]. Literature has revealed that turmeric (rhizomes of curcuma longa) when used as an antimicrobial agent has been very effective against gram-positive and gram-negative bacteria that are known to cause infections such as pneumonia, skin diseases, urinary tract infections, and meningitis in human beings [12-14]. Very few studies have been carried out with turmeric solution against endodontic infections. Hence, this study was done to compare the anti-bacterial effectiveness of conventional irrigants such as 5% sodium hypochlorite and 2% Chlorhexidine, along with a novel potential irrigant at two different concentrations, 6% and 9% turmeric solution on Fusobacterium nucleatum biofilm.

## Materials and methods

An isolate of Fusobacterium nucleatum ATCC 25586 (Microbiologies MN) was obtained. Brain heart infusion broth (BHIB) (HiMedia Laboratories, India) was used to prepare broth cultures for the biofilm experiment. Sterile glass microscope slides were immersed in 35 ml of BHIB in sterile centrifuge tubes. These tubes were later inoculated with 2.5 ml of the broth and were further incubated for 96 hours at 37°C. Thus, the biofilm development was seen in the prepared biofilm model.

The irrigants used in this study were Group I - 2% Chlorhexidine (Ammdent, India); Group II - 5% Sodium Hypochlorite (Prime Dental, Thane); Group III - 6% turmeric solution; Group IV - 9% turmeric solution and Group V - sterile distilled water. Group I, II, and V were commercially available. Group III and IV were prepared. Turmeric solution was obtained from the curcuma longa rhizomes by maceration method and rota evaporation was used to concentrate the extract. Concentrations of 6% and 9% turmeric solutions were obtained by mixing 6 and 9 grams of turmeric extract in 100 ml of sterile distilled water respectively. Sterile distilled water was used to wash carefully the glass slides with biofilm, which were removed from the centrifuge tubes. The slides with biofilm were then fully submerged in Petri dishes containing 35 ml of each of the five groups with 10 slides in each group for an exposure time of one minute and were immediately removed [15]. These slides were further dipped in Petri dishes containing neutralizing broth (Difco Laboratories, Michigan) supplemented with 0.5% Tween 20 (HiMedia Laboratories, India) to stop the antibacterial reaction of the irrigants. Following this, the slides were dipped in 10 ml of sterile BHIB in Petri dishes and biofilms were scraped from the surfaces of the slide with the help of a wire loop. The scraped biofilms were inoculated on brain heart infusion agar plates and incubated at 37°C for 96 hours in an anaerobic jar. The number of colony forming units in each plate was calculated to quantify the viable bacteria. Kruskal–Wallis test and Mann–Whitney U test using SPSS 15 software (IBM, Armonk, NY) were utilized to statistically analyse the data and to calculate statistically significant differences (p < 0.001).

## Results

The results were analyzed from the data obtained. A significant reduction of viable bacteria in the biofilm was seen in Groups I, II, III, and IV when compared to Group V (sterile distilled water). Group IV (9% turmeric solution) showed an overall statistically significant reduction (p < 0.001) of viable bacteria among all the groups. Group III (6% turmeric solution) showed a statistically significant reduction when compared to Group I (2% Chlorhexidine) and Group II (5% Sodium hypochlorite). Group II showed a greater reduction than Group I, however, it was not statistically significant between the two groups. Table 1 shows the significant bacterial reduction when 9% turmeric solution was used.

**Table 1:** Statistical analysis using Kruskal–Wallis test and Mann–Whitney U test. ***p < 0.001 **p < 0.01 Group I: Chlorhexidine (2%), Group II: Sodium hypochlorite (5%), Group III: Turmeric solution (6%), Group IV: Turmeric solution (9%), Group V: Sterile distilled water, cfu: colony forming unit.

Groups	log cfu/slide			H-value	p-value	Comparisons among the groups
	Mean	Std. deviation	Median			
Group I	3.441	0.031	3.447	46.026	<0.001	Group I vs Groups II, III, IV, V***
Group II	3.260	0.162	3.327			Group II vs Groups III, IV, V***
Group III	1.660	1.450	3.349			Group III vs Group IV**
Group IV	0.000	0.000	0.000			Group III vs Group V***
Group V	3.549	0.013	3.544			Group IV vs Group V***

## Discussion

The Fusobacterium nucleatum has the ability to form aggregates with other pathogenic bacteria in periodontal disease and it also acts as a connecting medium between early and late colonizers [6]. It is found in other body sites causing infections and was also found to be linked with severe forms of inter-appointment endodontic flare-ups [16]. Numerous studies have been done to find the microbes in the root canals and Fusobacterium nucleatum has been found in 44% of the cases [17]. Biofilm model was used in this study since bacteria are generally found in the root canal as biofilms and also because the behavior of bacteria in the biofilm is different from their planktonic counterparts [5]. The biofilm model used in this study had been previously used to test four antimicrobial irrigants against E. faecalis [15]. Many previous studies [5,15] on bacterial biofilms have used counting of colony forming units to obtain satisfactory results. Hence, the same methodology has been utilized in this study.

A study done by Williamson, et al. has shown sodium hypochlorite to exhibit statistically significant reduction in colony forming units at one minute of exposure [15]. Therefore, our study also utilized a similar type of exposure time for the various irrigants. In a pilot study done, 3% turmeric solution was found to be ineffective against E. faecalis. Hence, 6% and 9% concentrations of turmeric solution were used. In our study, it was found that 9% turmeric solution (Group IV) was significantly better in eradicating the biofilm than the other groups. There was no significant difference between 2% Chlorhexidine and 5% Sodium hypochlorite.

The active component in turmeric is called curcumin which is a polyphenolic compound found in it inherently. FtsZ, a prokaryotic homologue of eukaryotic cytoskeletal protein tubulin, polymerizes to form a Z-ring at the mid cell that brings about bacterial cell division. Rai, et al. found that curcumin increased the GTPase activity of FtsZ which disrupts the Z-ring formation and inhibits bacterial cytokinesis by inhibiting FtsZ assembly [18].

Sodium hypochlorite was effective in disrupting the biofilm to a certain level, which may be due to its tissue dissolving properties causing complete cellular dissolution [19]. But, the antibacterial activity of sodium hypochlorite in this study was not as statistically significant as turmeric solution. Studies comparing sodium hypochlorite and chlorhexidine, have found the former to be better than chlorhexidine in antimicrobial property [20-21]. Spratt, et al. found that sodium hypochlorite could not completely eradicate Fusobacterium nucleatum biofilm even after 15 minutes exposure [21]. In a study done by Clegg, et al., it was found that chlorhexidine killed bacteria but failed to disrupt biofilm [22]. In another study by Vitkov, et al., it was found that the efficiency of chlorhexidine was insufficient in causing biofilm disintegration [23].

As turmeric solution showed to be very effective against Fusobacterium nucleatum biofilm, further studies can be done on other microorganisms.

## Conclusions

Among the antimicrobial agents used in this study, 9% turmeric solution showed a significant bactericidal activity against Fusobacterium nucleatum biofilm. Thus, it has been found that turmeric solution is able to penetrate the biofilm and destroy Fusobacterium nucleatum and can hence be considered as an effective irrigant in endodontic therapy.
